# Development and Validation of an Educational Book on Self-Management in Inflammatory Bowel Disease Based on Patient Preferences and Expert Opinions: A Methodological Study

**DOI:** 10.3390/jcm12247659

**Published:** 2023-12-13

**Authors:** Narges Norouzkhani, Ali Bahari, Mahbobeh Faramarzi, Javad Shokri Shirvani, Saeid Eslami, Hamed Tabesh

**Affiliations:** 1 Department of Medical Informatics, Faculty of Medicine, Mashhad University of Medical Sciences, Mashhad 13944-91388, Iran or narges.norouzkhani@yahoo.com (N.N.);; 2 Department of Internal Medicine, Faculty of Medicine, Mashhad University of Medical Sciences, Mashhad 13944-91388, Iran; 3 Population, Family and Spiritual Health Research Center, Health Research Institute, Babol University of Medical Sciences, Babol 47176-47754, Iran; mahbob33@yahoo.com; 4 Department of Internal Medicine, Babol University of Medical Sciences, Babol 47176-47754, Iran; 5 Pharmaceutical Research Center, Mashhad University of Medical Sciences, Mashhad 13944-91388, Iran

**Keywords:** content validation studies, health educational materials, patient education, inflammatory bowel diseases, self-management

## Abstract

Background: Self-management education resources for inflammatory bowel disease (IBD) using concepts remain infrequent. We aim to describe the development and evaluation process of educational material for self-management in IBD based on patient preferences and expert opinions. Research design and methods: The method of this study includes two main phases of development and validation in five steps in the following order: (1) identification of information needs for patients with IBD; (2) content development with a comprehensive literature review and scientific texts related to IBD; (3) measuring the face validity of the content based on the expert opinions in the field of IBD; (4) validation of the content with the experts in the field of IBD; and (5) validation by target audiences. Results: The expert panel comprises ten gastroenterologists, nutritionists, psychologists, gynecologists, and nurses. The total suitability score is 79.5%. The final draft version of the educational self-management material was presented to 30 IBD patients who were satisfied (n = 24; 80%) with the material. Conclusions: This study shows the development process and is validated for face and content validity by the academic multidisciplinary expert panel and target group. Patients and their caregivers can use this content to cope with their disease.

## 1. Introduction

Inflammatory bowel disease (IBD) is defined as inflammation in the gastrointestinal tract with chronic or remitting/relapsing intervals without any definite cure [1,2]. Although it can start at any age, diagnosis is usually performed in the age range of 20–40 years old [3]. IBD possesses a heterogenous clinical presentation, such as abdominal pain, chronic diarrhea, rectal bleeding, and weight loss, in addition to some systemic symptoms, such as fatigue and fever [4], which affect all aspects of life quality [5]. Moreover, IBD imposes considerable morbidity and life-threatening complications, such as strictures, fistulas, infections, sclerosing cholangitis, blood clots, and cancer, all causing a significant burden on healthcare systems [6,7,8]. Although the exact cause of IBD is not fully elucidated, an abnormal intestinal immune response and altered gut microbiota are considered as the main culprits in genetically vulnerable individuals [1]. A disintegrated epithelial barrier of the intestine is thought to be a critical contributor to the initiation of IBD [9].

Based on the symptoms, disease location, and histopathological examination, IBD is classified into Crohn’s disease (CD) and ulcerative colitis (UC) [3]. 

The differentiation of UC from CD is a complicated issue. UC is characterized as a chronic inflammatory impairment that primarily begins from the rectal mucosa. The inflammation can be continued proximally, though with a sudden demarcation between inflamed and healthy regions. Periods of relapse and remission typically occur in patients with UC. CD is a much more intricate chronic inflammation with different manifestations in terms of age of onset, disease location, and behavior [10]. Discontinuous segments of injury (skip lesions), ileal involvement, and granulomatous inflammation are probable indicators of CD [11]. Although CD can occur in any part of the gastrointestinal tract, the small and large intestines are the risky parts [1]. 

In both UC and CD, T-cell-mediated responses are escalated. An amplified T-helper 1 and Th17 response trigger inflammation in CD, which results in the overactivation of certain proinflammatory cytokines, such as IL-17, IFN-γ, and TNF-α, leading to the exacerbation of the inflammation. In UC, a Th2-mediated response leads to the activation of B cells and natural killer T cells in a more efficient way that is implemented via IL-5 and IL-13 [12]. Intriguingly, susceptible genetic loci in IBD patients are associated with T-cell functions [13]. 

From 3.32 million estimated cases in 1990 to 4.90 million cases in 2019, IBD occurrence shows a global increase of 47.45%. Notably, the highest age-standardized prevalence rates were observed in high-income countries and those with high sociodemographic indices [14]. The prevalence and burden of IBD is greater in countries with a high index of development, and there is a clear trend in the prevalence of IBD from low to high SDI quintiles [8]. This shows the existence of specific environmental pressures and risk factors in these regions. These include, but are not limited to, urbanization, a higher level of hygienic environment, and consumption of high-meat and low-fiber diets [15,16]. In fact, living in a place with a high sociodemographic status increases the risk of IBD [8]. High sugar intake and smoking are among the other risk factors for decreased microbial species, which make individuals prone to IBD [17]. Although IBD is historically regarded as a disease of the Western world, there is a rapid increase in its incidence in other regions, including the Middle East, Asia, and South America, during the last two decades [18].

In these circumstances, patients with IBD are confronted with many concerns about the disease. They need technical information, especially about medications and the management of the disease [19]. Patients with IBD receive information from healthcare professionals (gastroenterologists and nurses), the Internet, and other sources, such as published articles and books, to gain sufficient knowledge. Having appropriate information is an effective tool for compliance [20]. 

### Aim

Therefore, it is necessary to evaluate patients’ information sources to effectively eliminate the gap in practical, understandable, and appropriate self-management education according to patient preferences. Considering these issues, we, for the first time, intend to develop and evaluate self-management educational material based on patient preferences, scientific evidence, and expert opinions.

## 2. Materials and Methods

### 2.1. Study Design

**Figure 1:**
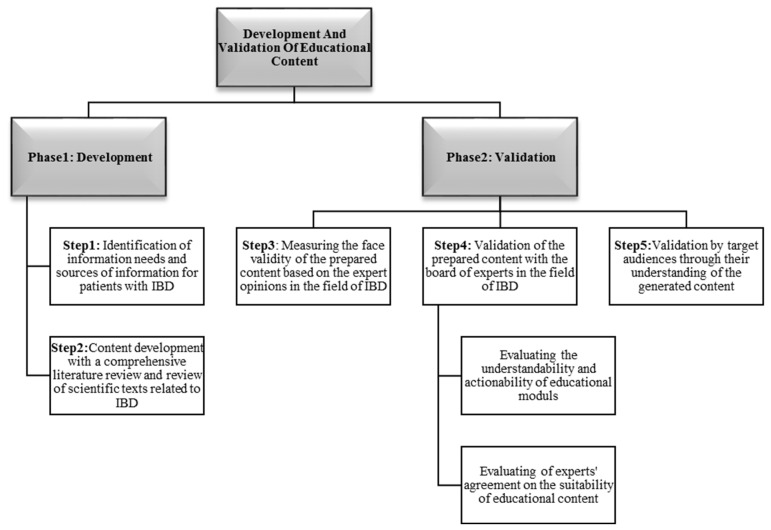
Steps followed in developing and validating the educational content.

The implementation method of this study included two main phases of development and validation of educational material in five steps in the following order: (1) identification of information needs and sources of information for patients with IBD; (2) content development with a comprehensive literature review and a review of the scientific texts related to IBD; (3) measuring the face validity of the prepared content based on the expert opinions in the field of IBD; (4) validation of the prepared content with the board of experts in the field of IBD; and (5) validation of the content by target audiences through their understanding of the generated content. The schematic diagram presented in Figure 1 shows the methodological steps.

### 2.2. Ethical Consideration 

This study was approved by the Research Ethics Committee of Mashhad Faculty of Medical Sciences in November 2021 (IR.MUMS.REC. 1400.230). All participants received and signed written informed consent before starting the study.

### 2.3. Identification of Information Needs and Sources of Information for Patients with IBD

Before compiling the content to determine the potential features of interest for the development of the educational content, the essential components and indicators that were effective in the thematic separation of the content were identified and extracted through a domain literature review. A literature search was conducted through electronic databases, including PubMed/Medline, CINAHL, APA PsycInfo, Psychology and Behavioral Sciences Collection, APA PsycArticles, ProQuest/ProQuest, and One Academic to find components between January 2000 and April 2022. The information was extracted and summarized in a table, and then, using the Delphi technique (3 rounds), essential and practical components of information were identified from the points of view of gastroenterology, nursing, and psychology experts. Finally, the results obtained from 3 rounds of Delphi in the form of a questionnaire whose validity and reliability were measured were given to patients with inflammatory bowel diseases to identify the essential and practical components of information from the patient’s point of view.

### 2.4. Content Development with a Comprehensive Literature Review and Review of Scientific Texts Related to IBD

The initial idea, framework, and justification for selecting educational topics were obtained based on collecting the required educational parameters based on the components considered by patients and specialists in the first step and from the review and aggregation of the headings of UC and Crohn’s guidelines. Then, the leading educational concepts needed were extracted from the review of scientific texts in the scientific databases. Finally, the draft of the educational material was prepared and printed by a research team, emphasizing official and up-to-date information about IBD based on the structure proposed by Duak and Shoemaker for the design of written health education materials [21,22].

### 2.5. Measuring the Face Validity of the Prepared Content Based on the Expert Opinions in the Field of IBD

After completing the initial draft based on the information obtained from the scientific texts and the results obtained from the previous steps, to check the qualitative face validity, the prepared educational material was provided to experts in the field of IBD. After careful reading, they were asked to examine the content regarding difficulty understanding words and phrases or possible ambiguities and using technical and specialized words.

### 2.6. Validation of the Prepared Content with the Board of Experts in the Field of IBD 

A multidisciplinary team of healthcare professionals in IBD patient care was selected and invited to participate in the study. The number of experts evaluating the content in this step exceeded those recommended by previous studies of at least five expert panels [23,24,25]. Convenience sampling was used to select expert judges. All experts with at least ten years of experience in teaching, studying, or treating patients with inflammatory bowel disease (IBD) and relevant health education experience or creating and evaluating educational material willing to participate in the study were enrolled. Those who did not respond to the questions within 14 days were excluded. After identifying the eligible expert, an informed consent form was provided to them by mentioning the 14-day evaluation period of study and content evaluation. The experts who agreed to participate received the educational material and two scales to evaluate the appearance and content of the material.

The evaluation tools used in this study included the Patient Education Materials Assessment Tool (PEMAT) and Suitability Assessment of Materials (SAM) tools. Educational modules were individually and rigorously evaluated with a patient education content evaluation checklist to measure understandability (17 questions) [26] and actionability (6 questions) using the AHRQ PEMAT-P user guide by gastroenterology subspecialists. “Understandability” was defined as the ability of people from different backgrounds with different health literacy abilities to understand educational concepts. In comparison, “actionability” is the ability of people to perform work based on the information from educational materials. All items had the answer options “agree” (1 point), “disagree” (0 points), and “not applicable” (questions that were not answered with “applicable” were excluded when calculating the score). The validity of this checklist was measured in a study in Iran, and its reliability was estimated to be 0.84 based on the Cronbach’s alpha test [27]. 

A panel of experts in related fields were asked to evaluate the content from the point of view of appropriateness. Appropriateness referred to whether the materials appropriately included adult learning principles and would lead to an increased understanding and positive behavior change [28,29]. The SAM assessed health content quality, difficulty, and ease with 22 group-wide items (content, literacy demand, graphics, layout, typography, learning simulation and motivation, and cultural appropriateness). The content was scored based on the existing criteria, and each item had a score of “excellent” (2 points), “adequate” (1 point), or “inadequate” (0 points). When an item did not apply to the content, the item was scored as “not applicable”, and the maximum score [22] for each non-applicable item was subtracted from the maximum score of 44 to form the denominator when calculating. The total SAM score was calculated by dividing the sum of the overall appropriateness scores for each factor by the total number of applicable items. This tool was translated in the research in Iran and tested for validity and reliability, the content validity index was determined at the level of the scale of the given tool at 0.9, and the reliability of the SAM tool was determined using a Cronbach’s alpha correlation coefficient of 0.95 [30].

### 2.7. Validation by Target Audiences through Their Understanding of the Generated Content 

Finally, after validating the content by a panel of experts, a group of target audiences was selected to evaluate the content with the stratified proportionate sampling method. The samples were divided into homogeneous subpopulations (UC and CD) based on the type of disease. The criteria for entering the patients were those who were being treated after suffering from inflammatory bowel diseases, were over 18 years old, literate, and were selected in three centers for gastrointestinal and liver diseases of adults. Additionally, patients in acute conditions of acute medical and psychiatric care were excluded from the study.

After identifying the eligible patients, an informed consent form was provided for them, which mentioned the 14-day evaluation period of study and content evaluation. The patients who agreed to participate received the educational material and a scale to evaluate the appearance and content of the material, including (1) organization; (2) writing style; (3) illustrations and layout; and (4) motivation [31,32], along with a guide on how to complete the scale, and a demographic information questionnaire was given to them. They also evaluated their overall level of satisfaction with the material on an 11-point numerical rating scale from completely dissatisfied (0) to completely satisfied (10). Patients also answered three questions: “In general, are you satisfied with the material?”, “How much did you like the content of the material?”, and “How much did the material help you understand the disease?”. They answered using a 5-point Likert scale ranging from 0 (completely dissatisfied) to 5 (completely satisfied).

### 2.8. Statistical Analysis

In this study, all data were collected by Microsoft Excel software, and the data analysis was performed with SPSS version 22 software.

#### 2.8.1. Measuring the Understandability and Actionability of the Content Based on Expert Opinions

A patient education content evaluation checklist was used to measure understandability and actionability. The total score was divided by the total possible scores, and the sum of understandability and actionability scores was calculated and presented as a percentage (%). A higher score indicated the content was understandable and actionable [26]. A score below 70% for PEMAT was related to the problematic understanding of concepts and non-actionability of the content. Our team targeted a score higher than 70% as an acceptable score, showing that the prepared content could be understood and applied [33,34]. For the final content validation analysis by experts, descriptive statistics, including central tendency and dispersion indices (mean and standard deviation) and frequency of data, were reported with numbers.

#### 2.8.2. Measuring Validity Index Measurements Based on Expert Opinions

The content validity index was calculated by experts using six items in SAM. This study used two content validity index (CVI) approaches at item and scale levels [35]. The item-level content validity index (I-CVI) was used to show the agreement between experts on the items, including the number of experts who gave each item a superior = 2 and adequate = 1 score divided by the total number of experts who provided a score. The scale-level content validity index/average calculation method (S-CVI/Ave) was calculated taking the sum of I-CVIs divided by the total number of items. In addition, the scale-level content validity/universal agreement (S-CVI/UA) was calculated at the scale level by adding all the items that obtained an I-CVI score equal to 1 and dividing this by the total number of items [36]. The minimum acceptable value of these indicators for educational materials with excellent content validity scores should be S-CVI/UA ≥ 0.8 or S-CVI/Ave ≥ 0.9 and I-CVI ≥ 0.78 for content evaluation. The degree of agreement and the reliability of the agreement between the experts were calculated using the inter-rater reliability using intra-class correlation (ICC). The agreement between experts about the content indicated its validity [37]. An ICC more significant than 0.8 indicated a solid agreement between experts.

#### 2.8.3. Validation Measurement Based on the Opinion of the Target Audience

To analyze the content validation by patients with inflammatory bowel diseases, descriptive statistics and frequency of data were reported with numbers and percentages. We considered this version to be sufficient if: (1) most raters (75% or more) had positive comments for the criteria; (2) the overall satisfaction rate was at least 7 out of 10; and (3) most experts (75% and more) were satisfied or completely satisfied for the satisfaction index in the questions measured with a Likert scale [38]. Finally, the number of agreements and the reliability of the agreements between the evaluations of the target group were calculated using the ICC.

## 3. Results 

### 3.1. Identification of Information Needs and Sources of Information for Patients with IBD 

Based on the literature review with the scoping review method [39], 56 information items were proposed for healthcare professionals during the Delphi sessions. Then, it was presented during the Delphi sessions with 57 healthcare professionals (mean age of 44.9 years old; standard deviation of 7.6), including adult gastroenterology and liver specialists, psychiatrists or clinical psychologists, and nursing faculty members. Most were male (56.1%), with an average work experience rate of 15.49 years. Finally, from the results obtained from 3 Delphi rounds, 45 essential information items were identified. These components were given to IBD patients in a questionnaire whose validity and reliability were measured to identify the essential information components from the patient’s point of view. Finally, 25 essential information items were determined based on the exploratory and confirmatory factor analysis. 

### 3.2. Content Development with a Comprehensive Literature Review and a Review of the Scientific Texts Related to IBD 

The draft of the educational pamphlet was prepared and printed by a six-person research team consisting of a medical informatics specialist, a clinical psychologist, an adult gastrointestinal and liver disease subspecialist, a pharmacist, and a biostatistician, in the form of 12 chapters. The total number of pages in each chapter was between 2 and 34 (Table 1).

### 3.3. Measuring the Face Validity of the Prepared Content Based on the Expert Opinions in the Field of IBD 

The initial draft was completed based on the information obtained from the scientific texts and the results of the previous steps. After completing the content, to check the formal and qualitative validity, the prepared educational material was provided to a five-member panel of experts consisting of adult gastrointestinal and liver disease faculty members (n = 3), clinical psychologist faculty members (n = 1), and nursing faculty members (n = 1). After careful reading, they were asked to examine the content regarding the difficulty in understanding words and phrases or possible ambiguities and using technical and specialized words. Then, after the correction, the final design and editing of the first version of the desired content were provided and expert opinions were applied to revise the content.

### 3.4. Validation of the Prepared Content with the Board of Experts in the Field of IBD 

Fifteen healthcare professionals in the field of IBD patient care were invited to review the first edition of the self-management material for IBD patients. Finally, ten experts consisting of a gastroenterologist (5 people), a nutritionist (1 person), a clinical psychologist (1 person), a gynecologist, childbirth and infertility (1 person), and an internal ward nurse (2 people) were selected. The experts on the panel consisted of six women (60%) and four men (40%) and had an average age of 55.6 years old (SD = 9.55) with an age range of 43 to 70 years old. Most of the experts were sub-specialists of gastrointestinal and liver diseases in adults (n = 5; 50%) and they were the faculty members of the University of Medical Sciences. The average experience of teaching or working in this field was 22 years (SD = 8.08), ranging from 10 to 37 years.

#### 3.4.1. Evaluating the Understandability and Actionability of Educational Content

All material modules were evaluated by five gastroenterologist experts with an average length of work experience of 18 years using the PEMAT tool from the points of view of understandability and actionability. The mean overall scores for understandability ranged from 83.63 to 97.5, and the mean overall scores for actionability ranged from 79.99 to 97.14 (Table 2).

#### 3.4.2. Evaluating the Suitability of Educational Content

After the experts considered the modifications, the modified content was presented to a multidisciplinary evaluator team to check the agreement between the experts. The experts were the same five gastroenterology and liver specialists and five experts from related fields. Then, according to the SAM criteria, all domains scored top marks. The percentage of grades varied from 71.6% to 88.3%. Among all the items, the lowest score of the SAM domain was associated with the domain of “learning simulation and motivation”, and the highest score of the SAM domain was related to “layout and typography”. Additionally, the content scored an average of 8.5 on a scale of 0 to 10 (as expressed in the SAM tool). Finally, the educational material obtained a score of 79.5%. These findings show that the educational material is rated “superior” according to the SAM ranking. The individually rated I-CVI for each item was significantly higher than the established percent agreement of 0.78. Additionally, the S-CVI/AVE between experts was 0.98, higher than 0, and the S-CVI/UA was equal to 0.81 and higher than 0.8 (Table 3).

#### 3.4.3. Examining the Agreement between Experts on Educational Material Using ICC

The Cronbach’s alpha coefficient in this study was 0.990, which indicated the high reliability of the data in this study. Four experts with an average work experience value of 15.75 (SD = 6.17) years presented the lowest average scores for the items (1.82). It seems that these judges are stricter than others. However, two judges with an average work experience score of 22.5 (SD = 2.5) years provided the highest average scores to the items. The reliability of the ICC for the average scores of experts and judges was 0.990, which indicated an excellent and robust agreement between the judges. Table 4 shows that the agreement among the raters for the material is statistically significant, *p* < 0.01. the agreement among all the experts on educational materials was solid, with an ICC ≥ 8.0 (Table 5).

### 3.5. Validation by Target Audiences through Their Understanding of the Generated Content 

The evaluation of the 2nd edition of the material was performed by 30 patients with IBD (UC = 15; CD = 15), including 19 women (63%) and 11 men (37%). They had an average age of 37.8 years old (SD = 11.27) with an age range of 20 to 68 years old. Most of them were in remission (n = 23; 76.67%). The average number of disease months was 133.77 (SD = 79.61), ranging from 7 to 264 months. Most evaluators had the Bachelor’s degree (n = 20; 67%). “Motivation” received the highest positive responses among the four rated domains. A total of 96.67% thought that every person who read this material would understand what it was about and that the prepared educational materials dealt with the critical issues about the disease so that patients with CD or UC could take proper care of themselves. A total of 90% of them were motivated to read the material to the end and believed that this material suggested that you thought about taking care of your digestive system and quality of life. Moreover, finally, they gave the material an overall average score of 8.47 (1.07). Overall, most patients were satisfied (80%) or very satisfied (20%) with the content of the material, and 100% of patients believed that the content helped them understand the disease (Table 6). In addition, patients’ suggestions about the content were included.

#### Examining the Agreement between IBD Patients on Educational Material Using ICC

The value of the Cronbach’s alpha coefficient in the evaluation of the target group was 0.99, which indicated the high reliability of the data in this study. The reliability of the ICC for the average evaluators of the target group was 0.998, which was considered to be an excellent and robust agreement of the target group with each other. 

## 4. Discussion

This study aimed to improve the awareness of patients with IBD by creating educational material according to their preferences and based on the scientific evidence and specialists’ opinion. The present study combined different aspects of lifestyle and disease. All educational modules were developed based on the needs and interests of IBD patients and healthcare providers in this field. The development of modules in this way empowers IBD patients in terms of adapting to and managing the disease, and finally improves their quality of life. Previous studies confirmed the importance of education and increasing the awareness of these patients to improve their quality of life [40,41]. 

The present study used three evaluation methods: PEMAT, SAM, and examining patients’ perspectives. The PEMAT questionnaire was a systematic method that evaluated the two essential domains of understandability and actionability, in which scores of 70% and below were considered poor performances [26]. Overall, the mean module scores for understandability ranged from 83.63 to 97.5, and the mean overall scores for actionability ranged from 79.99 to 97.14, indicating that most module units were highly acceptable among healthcare professionals in this study. Understandability scores indicated that the module was easy to understand and contained explicit content. Folk words were used instead of medical terms; if there were medical terms, their meanings and subtitles were presented. Appropriate visual aids were also used in the module.

Additionally, module actionability scores indicated the module’s explicit explanation of enforceable actions. Moreover, the expert panel considered a total content assessment with the SAM tool adequate in all domains (content, objectives, language, images, design, motivation, and cultural setting). Overall, the content was ranked very high, with 79.5% points. The content validation process in this study was consistent with other studies [32,36]. However, two other studies used a 4-point Likert scale versus the SAM instrument [42]. The Delphi technique [37] used the binomial test and Kappa to determine concordance in expert ratings. In all studies, experts, judges, or specialists were involved in the validation process due to understanding the content [43]. 

The experts in this study were the beneficiaries of IBD education, and the agreement between them was the main focus of the content validation. Inter-rater agreement was measured using S-ICVI/UA. The result obtained for educational material is higher than 0.8, which indicates an excellent agreement. Such a process was similarly used in a previous study regarding the material on depression during pregnancy [36]. In the present study, the ICC determined the reliability between the raters, and the achievement of a correlation coefficient of higher than 0.8 in their agreement on the adequacy of the educational material indicated a high correlation coefficient. 

Evaluating educational material by target users (IBD patients) is essential to assessing experts. Since educational material is made for user consumption, the expert validation process will be pointless if the target users believe that the content is inappropriate. [36,37]. In the present study, educational rating by target users and experts was not performed by the same tool, since the research team found it difficult for users to use PEMAT and SAM tools. Therefore, a checklist adapted from previous studies was provided for the users, which consisted of simple and understandable questions [31,32,44]. In this study, only one patient (3.33%) was not interested in the material’s content. Individual factors, such as type of illness, duration of illness, age, gender, and level of education, were investigated in this study, and it seemed that the amount of knowledge and awareness of the patient that may help them understand self-management education was substantial.

It should be noted that, according to the research team, the present study was the first study in the world that developed educational material for IBD patients systematically to eliminate the existing gap in practical, understandable, and appropriate self-management education; take into account the preferences of the patient; and evaluate it with a combined approach based on the views of the experts and patients. Therefore, evaluating patients’ information sources is necessary to effectively fill in the gap in practical, understandable, and appropriate self-management education according to patient preferences. 

### 4.1. Strengths and Limitations 

The study’s main strength was developing educational material in the form of self-management with an interactive language to provide practical tips for adults with different educational backgrounds. In addition, this book can be effectively used in clinical and research settings to solve the daily lifestyle problems of adults with chronic IBD and answer their various questions about adaptation to IBD. However, due to IBD’s active state and relapse, it should be considered that a complex health condition usually requires a comprehensive and multidisciplinary approach with psychological, nutritional, and pharmacological interventions. 

The present study had some limitations. The book was written in Persian and the authors used this Persian version to conduct a study on a sample of Iranian patients without cross-cultural validations of the English version of the book.

Moreover, the effectiveness of the self-management book in improving participants’ knowledge or changing their disease management behaviors was not evaluated. Because of these limitations, the self-management book must be localized and adapted to different countries’ languages and cultural characteristics. Although this study provided educational material about IBD in the form of a book, the best way to deliver educational material is unknown at present. Researchers can also use tools, such as electronic materials, games, and video clips, to provide educational material to target populations.

### 4.2. Recommendations for Future Research 

This book, as an up-to-date and comprehensive educational content, has been developed to increase awareness and empower and improve the quality of life in IBD patients. For this reason, this content can be used in IBD clinics for patient education. Additionally, researchers who intend to create educational materials in Iran can use this validation process to produce relevant and local educational content. 

## 5. Conclusions

The present study shows that self-management educational materials in IBD patients are valid in terms of content and appearance based on high evaluation scores by experts. In addition, the target users also had the opportunity to participate in the development and validation process. Therefore, the involvement of different evaluators creates a powerful tool for improving patients’ self-management practices. A clinical trial to evaluate the effectiveness of this educational material in changing behavior according to the use of educational instructions is being planned and designed by the research team of this study.

## Figures and Tables

**Table 1:** Summary of educational material developed for self-management of patients with IBD.

Main Headlines	Details (Samples)	Number of Pages
Chapter 1: “Overview of IBD”	The structure and function of the digestive system General IBD Extraintestinal symptoms and complications Factors affecting the recurrence and flare-up of disease symptoms When to visit or call a doctor for IBD	25
Chapter 2: “Diagnostic methods”	Types of diagnostic methods	10
Chapter 3: “Pharmaceutical and non-pharmacological management of IBD”	Drug treatment of IBD (side effects, method of administration, duration of response, necessary precautions during administration, necessary tests during administration, vaccination, essential drug interactions, and effects on fertility, pregnancy, and breastfeeding) Intestinal surgery in disease	30
Chapter 4: “Sexual relations, fertility, and issues of women with IBD”	Women with IBD (menstruation and menopause) Sexual and emotional relationships of pregnancy and conception of women and men with IBD Childbirth, breastfeeding, and vaccination of babies with mothers suffering from IBD	12
Chapter 5: “Intestinal cancer in people with IBD”	Colon cancer risk factors and symptoms Recommendations to reduce the risk of colon cancer	3
Chapter 6: “ Cosmetic procedures in IBD”	Skin, hair, and beauty	2
Chapter 7: “Non-IBD medications”	Safe/Unsafe drug in IBD	3
Chapter 8: “Infections and viral infectionsdiseases in IBD”	viral infections and management	4
Chapter 9: “Complementary medicine”	Types of traditional, complementary medicine methods	1
Chapter 10: “Nutrition and nutritional supplements in IBD”	Diet	34
Chapter 11: “Preventive care in IBD”	Preventive and general healthcare in IBD patients	6
Chapter 12: “Lifestyle in IBD”	Management of fatigue in patients with IBD Mental problems and therapeutic solutions to deal with mental problems Exercise and physical activity in patients with IBD Travel recommendations for IBD Compatibility with IBD Fasting recommendations for IBD	25

**Table 2:** Understandability and actionability of printed educational material developed for self-management of patients with IBD.

Main Headlines	Understandability (%)			Actionability (%)		
	Max	Min	Mean (SD)	Max	Min	Mean (SD)
Chapter 1: “Overview of IBD”	34.12	82.35	89.41 (SD = 4.92)	100	83.33	93.33 (SD = 9.13)
Chapter 2: “Diagnostic methods”	100	82.35	95.29 (SD = 7.67)	100	87.71	97.14 (SD = 6.39)
Chapter 3: “Pharmaceutical and non-pharmacological management of IBD”	94.12	88.24	90.59 (SD = 3.22)	100	85.71	94.28 (SD = 7.82
Chapter 4: “Sexual relations, fertility, and issues of women with IBD”	94.12	82.35	85.88 (SD = 5.26)	100	85.71	94.28 (SD = 7.82)
Chapter 5: “Intestinal cancer in people with IBD”	100	92.31	96.92 (SD = 4.21)	100	83.33	93.33 (SD = 9.13)
Chapter 6: “Cosmetic measures for IBD”	100	91.67	96.66 (SD = 4.56)	100	83.33	89.99 (SD = 9.13)
Chapter 7: “Other medications in patients with IBD”	100	93.75	97.50 (SD = 3.42)	100	85.71	94.28 (SD = 7.82)
Chapter 8: “Viral and infectious diseases in IBD patients”	94.12	88.24	91.76 (SD = 3.22)	100	85.71	97.14 (SD = 6.39)
Chapter 9: “Complementary medicine”	90.91	72.73	83.63 (SD = 7.60)	85.71	71.43	79.99 (SD = 7.82)
Chapter 10: “Nutrition and nutritional supplements in IBD patients”	94.12	88.24	92.94 (SD = 2.62)	100	85.71	97.14 (SD = 6.39
Chapter 11: “Preventive care in IBD patients”	100	86.67	93.33 (SD = 4.71)	100	85.71	94.28 (SD = 7.82)
Chapter 12: “Lifestyle in IBD patients”	94.12	76.47	88.23 (SD = 8.32)	100	71.43	85.71 (SD = 10.10)

**Table 3:** Scoring and agreement of experts.

Main Factors	Items	Appropriate	Somewhat Appropriate	Inappropriate	I-CVI	Mean (SD)
Content	Purpose is evident	10	0	0	1	6.40 (SD = 1.53)
	Content about behaviors	6	4	0	1	
	Scope is limited	5	5	0	1	
	Summary or review included	3	7	0	1	
Literacy demand	Reading grade level	0	9	1	0.9	7.60 (SD = 1.97)
	Writing style, active voice	8	2	0	1	
	Vocabulary uses common words	5	5	0	1	
	Context is given first	5	4	1	0.9	
	Learning aids via “road signs”, subtitles, and captions	10	0	0	1	
Graphics	Cover graphic shows purpose	3	6	1	0.9	8.30 (SD = 2.05)
	Type of graphics	10	0	0	1	
	Relevance of illustrations	7	3	0	1	
	Lists and tables explained	8	2	0	1	
	Captions used for graphics	6	4	0	1	
Layout and typography	Layout factors	4	6	0	1	5.30 (SD = 0.84)
	Typography	9	1	0	1	
	Subheads (“chunking”) used	10	0	0	1	
Learning simulation and motivation	Interaction used	3	6	2	0.9	4.30 (SD = 1.68)
	Behaviors are modeled and specific	5	5	0	1	
	Motivation, self-efficacy	6	4	0	1	
Cultural appropriateness	Match in logic, language, and experience (LLE)	10	0	0	1	3.10 (SD = 0.32)
	Cultural images and examples	1	9	0	1	
Total SAM score (%)	79.50					
S-CVI/Ave	0.98					
S-CVI/UA	0.82					

**Table 4:** Intraclass correlation coefficient between the rating of experts of educational material.

	Intraclass Correlation ^b^	95% Confidence Interval		F-Test with True Value 0			
		Lower Bound	Upper Bound	Value	df1	df2	Sig
Single Measures	0.906 ^a^	0.846	0.952	97.454	22	198	0.000
Average Measures	0.990	0.982	0.995	97.454	22	198	0.000

Two-way random effects model where both people effects and measures effects are random. ^a^. The estimator is the same, whether the interaction effect is present or not. ^b^. Type C intraclass correlation coefficients using a consistency definition. The between-measure variance is excluded from the denominator variance.

**Table 5:** Item correlation matrix between the rating of experts on educational material.

	E1	E2	E3	E4	E5	E6	E7	E8	E9	E10
E1	1.000	0.921	0.949	0.891	0.888	0.946	0.944	0.948	0.964	0.932
E2	0.921	1.000	0.856	0.941	0.945	0.920	0.919	0.911	0.961	0.890
E3	0.949	0.856	1.000	0.827	0.883	0.892	0.919	0.905	0.920	0.939
E4	0.891	0.941	0.827	1.000	0.916	0.942	0.937	0.860	0.931	0.905
E5	0.888	0.945	0.883	0.916	1.000	0.907	0.919	0.886	0.917	0.926
E6	0.946	0.920	0.892	0.942	0.907	1.000	0.956	0.907	0.942	0.949
E7	0.944	0.919	0.919	0.937	0.919	0.956	1.000	0.861	0.954	0.946
E8	0.948	0.911	0.905	0.860	0.886	0.907	0.861	1.000	0.901	0.888
E9	0.964	0.961	0.920	0.931	0.917	0.942	0.954	0.901	1.000	0.922
E10	0.932	0.890	0.939	0.905	0.926	0.949	0.946	0.888	0.922	1.000

**Table 6:** Grading of educational material by IBD patients.

Items	Number of Positive Responses (%)	Number of Negative Responses (%)	Number of without Comment (%)
Organization (yes/no/do not know)			
Did the cover catch your attention?	21 (70.00)	8 (26.67)	1 (3.33)
Is the content sequence adequate?	17 (57.00)	13 (43.00)	0 (0)
Is the structure of the material neat and organized?	17 (57.00)	11 (36.67)	2 (6.67)
Writing style (easy/hard/do not know)			
How was the understanding of the sentences for you?	28 (93.33)	0 (0)	2 (6.67)
How was the content in general?	25 (83.33)	4 (13.33)	2 (6.67)
How was the text?	30 (100)	0 (0)	0 (0)
Illustrations and layout (simple/complicated/do not know)			
How were the Illustrations?	27 (90.00)	3 (10.00)	0 (0)
Do the illustrations complement the text?	24 (80.00)	4 (13.33)	2 (6.67)
Do the pages or sections look organized?	25 (83.33)	5 (16.67)	0 (0)
Motivation (yes/no/do not know)			
Do you think any Crohn’s or ulcerative colitis patient who reads this will understand what this material is about?	29 (96.67)	1 (3.33)	0 (0)
Did you feel motivated to read the material to the end?	27 (90.00)	1 (3.33)	2 (6.67)
Does the educational material address the necessary issues about the disease so that patients with Crohn’s or ulcerative colitis can perform appropriate self-management?	29 (96.67)	0 (0)	1 (3.33)
Does the educational material encourage you about disease management and quality of life?	27 (90.00)	2 (6.67)	1 (3.33)
Total agreement score (%)	83.64		

## Data Availability

The original data presented in the study are included in the article; the data supporting the results of the present study are only available from the authors upon reasonable request. Data are not publicly available.
